# Accelerated large volume irradiation with dynamic Jaw/Dynamic Couch Helical Tomotherapy

**DOI:** 10.1186/1748-717X-7-191

**Published:** 2012-11-12

**Authors:** Sonja Krause, Sebastian Beck, Kai Schubert, Steffen Lissner, Susanta Hui, Klaus Herfarth, Juergen Debus, Florian Sterzing

**Affiliations:** 1 Department of Radiation Oncology, University Hospital Heidelberg, INF 400, 69120, Heidelberg, Germany; 2 Department of Therapeutic Radiology, University of Minnesota, 420 Delaware St. SE, Mayo Mail Code 494, Minneapolis, , MN 55455, USA

**Keywords:** Dynamic jaw/dynamic couch, Helical tomotherapy, Large volumes, Hemithoracic irradiation, Whole abdominal irradiation, Total marrow irradiation

## Abstract

**Background:**

Helical Tomotherapy (HT) has unique capacities for the radiotherapy of large and complicated target volumes. Next generation Dynamic Jaw/Dynamic Couch HT delivery promises faster treatments and reduced exposure of organs at risk due to a reduced dose penumbra.

**Methods:**

Three challenging clinical situations were chosen for comparison between Regular HT delivery with a field width of 2.5 cm (Reg 2.5) and 5.0 cm (Reg 5.0) and DJDC delivery with a maximum field width of 5.0 cm (DJDC 5.0): Hemithoracic Irradiation, Whole Abdominal Irradiation (WAI) and Total Marrow Irradiation (TMI). For each setting, five CT data sets were chosen, and target coverage, conformity, integral dose, dose exposure of organs at risk (OAR) and treatment time were calculated.

**Results:**

Both Reg 5.0 and DJDC 5.0 achieved a substantial reduction in treatment time while maintaining similar dose coverage. Treatment time could be reduced from 10:57 min to 3:42 min / 5:10 min (Reg 5.0 / DJDC 5.0) for Hemithoracic Irradiation, from 18:03 min to 8:02 min / 8:03 min for WAI and to 18:25 min / 18:03 min for TMI. In Hemithoracic Irradiation, OAR exposure was identical in all modalities. For WAI, Reg 2.5 resulted in lower exposure of liver and bone. DJDC plans showed a small but significant increase of ∼ 1 Gy to the kidneys, the parotid glans and the thyroid gland. While Reg 5.0 and DJDC were identical in terms of OAR exposure, integral dose was substantially lower with DJDC, caused by a smaller dose penumbra.

**Conclusions:**

Although not clinically available yet, next generation DJDC HT technique is efficient in improving the treatment time while maintaining comparable plan quality.

## Background

Helical Tomotherapy (HT) has proven to have unique capacities for the radiotherapy treatment of large target volumes [1,2]. Technical details of HT have been described extensively before [3]. In brief, a HT unit with its ring gantry forms a fusion of a linear accelerator and a helical CT scanner [4]. The treatment beam can be shaped into different beam widths depending on the opening angle of the secondary collimator (jaws). In an inverse treatment planning process, the MLC conformation is optimised to obtain highly conformal radiation doses to the target [5]. As the patient is moved through the gantry, targets up to a length of 160 cm can be treated in one treatment run. While HT can yield excellent target coverage and sparing of organs at risk in the treatment of very large volumes [6], it has often been criticised because of long treatment times.

During the last years, other techniques of rotational IMRT have evolved. As initially described by Otto [7], in volumetric modulated arc therapy dose is delivered by a modulated beam with a field size of up to 40 × 40 cm using one or very few rotations. This technique offers very fast IMRT treatments, especially for target volumes of low or intermediate complexity [8]. Satisfactory coverage of complex volumes frequently needs more than one rotation, and very large target extensions often demand multiple isocenters that require additional patient setup time.

With HT, the problem of field junctioning in large targets has been solved by the helical superposition of multiple beam rotations. However, treatment time is still a crucial factor influencing treatment safety, as patient motion and the subsequent risk of dose uncertainties increases with treatment time. New HT developments such as Dynamic Jaw/Dynamic Couch (DJDC) have the potential to speed up treatment substantially: While in current HT the field width (determined by the jaw opening) as well as the couch speed remain constant once chosen at the beginning of treatment planning, DJDC allows for the dynamic adaptation of field width and couch speed depending on target complexity. Thus, areas with less complex target shapes can be treated fast and with a broad field width without compromising OAR sparing and target coverage in more critical localisations [9,10]. In addition, dynamic opening of the jaws at the start and end of the target reduces the craniocaudal dose penumbra caused by a constant field width. Currently, DJDC is still in development and not available for clinical routine yet.

To put DJDC to the test, we chose three clinical settings where the radiation oncologist is faced with the challenge of treating very large and complex volumes in close proximity to critical organs at risk. The first was the treatment of one complete hemithorax after radical surgery for Malignant Pleural Mesothelioma. Secondly, we investigated Whole Abdominal Irradiation (WAI) after surgery for ovarian cancer, and thirdly the treatment of the complete bone marrow (Total Marrow Irradiation, TMI) as part of conditioning regimens before stem cell transplantation.

The present planning study compares state-of-the-art HT with 2.5 cm and 5.0 cm field width to DJDC and characterises the benefits of future HT developments for the treatment of three very large volumes: Hemithoracic Irradiation, WAI and TMI.

## Materials and methods

This plan comparison study consisted of 15 anonymised clinical cases forming three groups: 5 patients with Malignant Pleural Mesothelioma had obtained a hemithoracic irradiation with HT and 5 patients had been treated with a HT Whole Abdominal Irradiation (WAI) for ovarian cancer FIGO IIIc. In preparation for a clinical Total Marrow Irradiation (TMI) trial, the plans for TMI were conducted on 5 planning CT scans of patients that had obtained a craniospinal irradiation with a suitable CT scan length. For all settings, the original CT scans, and for hemithoracic irradiation and WAI also the original structure sets were used to generate Reg 2.5, Reg 5 and DJDC plans on the original data sets. All plans were calculated and evaluated by the same two persons to keep variations in planning procedure als low as possible.

### Hemithoracic irradiation

Two patients suffered from left-sided, three patients from right-sided mesothelioma. All 5 patients had obtained neoadjuvant chemotherapy with cisplatin and pemetrexed and extrapleural pneumonectomy and had been treated with regular HT Hemithoracic irradiation using a field width of 2.5 cm (Reg 2.5), because in clinical routine, a 5.0 cm field width resulted in larger exposure of the neck as well as the left kidney or the liver. Planning CT scans with 3.0 mm slices were used. The PTV included the former pleural cavity and the surgical scars with a security margin of 1.0 cm and had an average volume of 3199 ml. A median dose of 54.0 Gy in 27 fractions was prescribed to the PTV. Reg 2.5 plans were compared to Reg 5.0 and DJDC 5.0 plans in terms of treatment time, target coverage and exposure of organs at risk (OAR). All HT modalities were planned on a fine calculation grid with a pitch of 0.287. For Reg 2.5 and Reg 5.0, the Intensity Modulation Factor (IMF; maximum leaf intensity divided by the average leaf intensity) was increased gradually up to 2.4, while for DJDC an IMF of 2.2-2.5 was reached. Of note, the IMF for DJDC represents the value given during planning, not the actual IMF calculated by the planning system, as the research version does not allow the specification of a commissioned treatment machine and consecutively does not permit a final dose calculation.

### Whole abdominal irradiation

All 5 patients were treated for ovarian cancer stage FIGO IIIc after resection of the ovaries and the peritoneum including all macroscopic tumor lesions and adjuvant platinum-based chemotherapy. The PTV consisted of the complete peritoneal cavity with a security margin of 1.5 cm (2.5 cm in craniocaudal direction). For HT planning purposes, the PTV was subdivided in order to control underdosage due to OAR sparing: Two PTVs surrounded the left and right kidneys, one covered the upper part of the abdomen close to the lung and the fourth PTV covered the rest of the abdominal cavity. Planning CT scans of 5.0 mm slice thickness were used. A median dose of 30.0 Gy in 1.5 Gy fractions was prescribed. Again, the Reg 2.5 plans used in clinical routine were compared to Reg 5.0 and DJDC 5.0 plans. Both HT plans had a pitch of 0.287; Reg 2.5 and Reg 5.0 plans needed an IMF of 4.0 while DJDC planning resulted in an IMF of 3.6 - 4.0.

### Total marrow irradiation

In preparation for clinical phase I TMI trial, planning CT scans with 5 mm slice thickness of patients receiving craniospinal irradiation were chosen for TMI planning because of the suitable scan length. The CTV comprised the complete bone marrow with the exception of the mandible and maxillary bones for a better sparing of the oral mucosa. For PTV construction, different security margins were applied depending on localisation: For the skull down to C2, no security margin was applied as the fixation with a rigid mask ensures precise positioning, on other locations, a security margin of 5 mm was chosen. A median of 18 Gy in 9 fractions was prescribed to the PTV. As for the other indications, Reg 2.5 was compared to Reg 5.0 and DJDC 5.0. To account for the large craniocaudal extension of the PTV, a pitch of 0.43 was chosen for HT plans; the IMF was increased up to 2.5 for all HT plans.

### Plan comparison and statistical analysis

Plan quality concerning the PTV was judged using D_99_ and D_1_ (dose to 99% and 1% of the target volume), CI_95_ (Conformity Index; total volume covered by the 95% isodose divided by the volume of the PTV covered by the 95% isodose), UI (Uniformity Index; dose covering 5% of the PTV divided by the dose covering 95% of the PTV) and TV_95%_ (PTV volume covered by 95% of the prescribed dose divided by the PTV volume). Sparing of organs at risk was judged by maximum and average dose as well as integral dose (product of the total volume and the mean dose to that volume).

For statistical analysis, a two-sided paired t-test was used. A value of p < 0.05 was considered statistically significant.

## Results

### Treatment time

**Figure 1:**
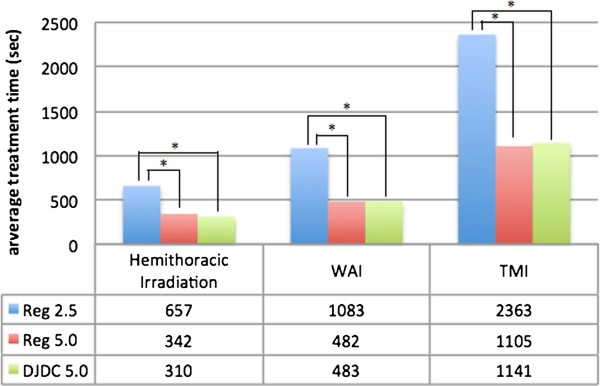
**Average treatment times (sec) for Hemithoracic Irradiation, Whole Abdominal Irradiation (WAI) and Total Marrow Irradiation (TMI) for the Reg. 2.5, Reg 5.0 (“regular” delivery with a 2.5 cm and 5.0 field width) and DJDC 5.0 (Dynamic Jaw/Dynamic Couch delivery with a 5.0 cm maximum field width) plans.** In all three plan groups, DJDC 5.0 came up with significantly shorter treatment times compared to Reg 2.5 (* p < 005).

Regular HT with 2.5 cm field width of all three types of complex and very large target volumes took an average of 10:57 min (Hemithoracic Irradiation), 18:03 min (WAI) and 39:23 min (TMI). Using a field width of 5.0 cm in regular delivery more than halved average treatment time to 3:42 min for Hemithoracic Irradiation, 8:02 min for WAI and 18:25 min for TMI. With DJDC technique, Hemithoracic Irradiation took longer than with Reg 5.0 (5:01 min, not significant), while WAI and TMI treatments were as fast as with Reg 5.0 (8:03 min and 19:01 min) (see Figure 1). While treatment speed was comparable for DJDC and Reg 5.0, the latter produced a larger craniocaudal dose penumbra and consecutively a higher integral dose (see below).

### Target coverage

**Figure 2:**
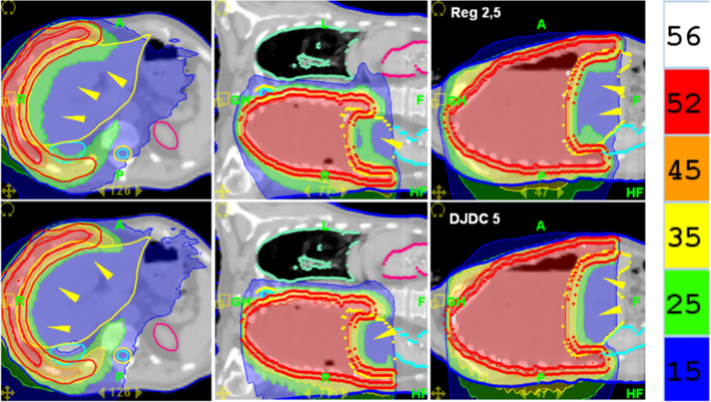
**Hemithoracic Irradiation: Dose distributions for Reg 2.5 (“regular” delivery with a 2.5 cm field width; upper row) and DJDC 5.0 (Dynamic Jaw/Dynamic Couch delivery with a 5.0 cm maximum field width; lower row).** Note the sparing of the liver in both modalities (arrowheads) and the reduced craniocaudal dose penumbra in DJDC plans.

**Figure 3:**
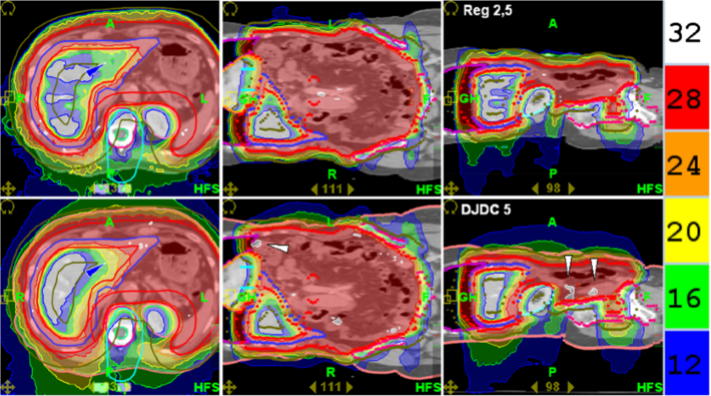
**Whole Abdominal Irradiation: Dose distributions for Reg 2.5 (“regular” delivery with a 2.5 cm field width; upper row) and DJDC 5.0 (Dynamic Jaw/Dynamic Couch delivery with a 5.0 cm maximum field width; lower row).** Note the slightly worse lateral dose penumbra in DJDC 5, for example in the left lobe of the liver (dark arrowhead) and the increased incidence of hotspots in DJCD (light arrowheads).

**Figure 4:**
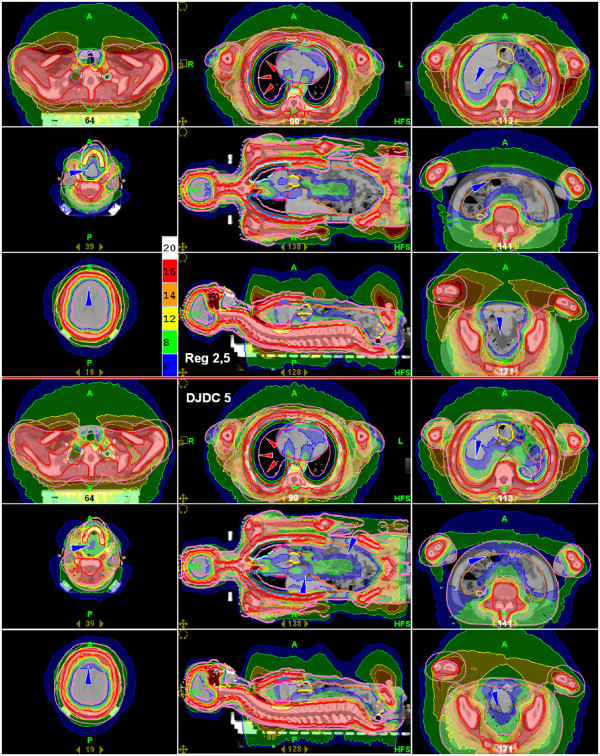
**Total Marrow Irradiation: Dose distributions for Reg 2.5 (“regular” delivery with a 2.5 cm field width; upper half) and DJDC 5.0 (Dynamic Jaw/Dynamic Couch delivery with a 5.0 cm maximum field width; lower half).** Note the equivalent lung sparing in both modalities (light arrowheads), which is accompanied by a more severe dose fall-off in the thoracic wall in DJDC 5.0. The dark arrowheads indicate areas of higher dose exposure in DJDC 5.0 that form the correlate for the non-significantly increased integral dose in DJDC 5.0.

Although for DJDC planning a maximum field width twice as large as in regular delivery was permitted, dose distribution in the target volumes remained on a similar level (see Tables 1, 2, 3 and Figures 2, 3, 4). Target coverage as indicated by TV95% was almost identical in all modalities for Hemithoracic Irradiation and TMI. For WAI, a significantly better coverage could be attained with Reg 2.5.

**Table 1:** Hemithoracic irradiation: plan characteristics for Reg 2.5, Reg 5.0 (“regular” delivery with a 2.5 cm and 5.0 cm field width) and DJDC 5.0 (dynamic jaw/dynamic couch delivery with a 5.0 cm maximum field width)

		**Reg 2.5**	**Reg 5.0**	**DJDC 5.0**
PTV	D_99_ (Gy)	48.6 ± 0.6	49.5 ± 1.0	48.7 ± 0.6
	D_1_ (Gy)	56.0 ± 0.5	57.7 ± 1.3	55.9 ± 0.2
	CI_95_	1.14 ± 0.08	1.19 ± 0.06	1.16 ± 0.05
	UI	1.06 ± 0.03	1.09 ± 0.04	1.06 ± 0.00
	TV_95%_	0.967 ± 0.015	0.975 ± 0.009	0.967 ± 0.004
liver	mean (Gy)	20.1 ± 9.1	20.7 ± 9.4	19.9 ± 10.1
ipsilateral kidney	mean (Gy)	8.8 ± 3.0	8.3 ± 2.9	8.4 ± 2.6
contralateral kidney	mean (Gy)	4.0 ± 1.2	3.6 ± 0.9	3.3 ± 1.2
contralateral lung	mean (Gy)	6.1 ± 0.6	6.2 ± 0.7	6.0 ± 1.0
spinal cord	max (Gy)	35.0 ± 6.2	32.3 ± 0.9	34.1 ± 2.1
heart	mean (Gy)	26.5 ± 4.8	26.9 ± 6.9	26.7 ± 5.3
esophagus	mean (Gy)	32.0 ± 3.2	32.5 ± 1.2	31.5 ± 3.1
integral dose	(Gy x l)	411.4 ± 24.5	513 ± 110.9	414.6 ± 31.3

No statistically significant differences could be detected in dose distribution in the PTV and sparing of organs at risk.

**Table 2:** Whole abdominal irradiation: plan characteristics for Reg 2.5, Reg 5.0 (“regular” delivery with a 2.5 cm and 5.0 cm field width) and DJDC 5.0 (dynamic jaw/dynamic couch delivery with a 5.0 cm maximum field width)

		**Reg 2.5**	**Reg 5.0**	**DJDC 5.0**
total PTV	CI_95_	1.11 ± 0.08	1.13 ± 0.04	1.16 ± 0.03
	TV_95%_	**0.925 ± 0.013**	**0.887 ± 0.007**	**0.931 ± 0.022**
right kidney	mean (Gy)	11.9 ± 0.8	12.3 ± 1.1	13.0 ± 0.9
left kidney	mean (Gy)	10.7 ± 0.8	11.7 ± 1.0	11.5 ± 0.8
bone	mean (Gy)	**10.8 ± 1.1**	**12.9 ± 1.0**	11.7 ± 0.8
spinal cord	max (Gy)	12.7 ± 2.7	12.5 ± 1.1	13.6 ± 2.7
heart	mean (Gy)	9.5 ± 2.4	10.6 ± 2.7	10.5 ± 1.5
liver	mean (Gy)	**22.3 ± 0.9**	**23.5 ± 0.5**	**23.7 ± 0.9**
integral dose	(Gy x l)	**369.3 ± 67.8**	**496.2 ± 92.3**	**382.9 ± 52.4**

Target coverage and bone and liver sparing were significantly better with Reg 2.5 (bold print). Reg 5.0 resulted in a significantly higher integral dose than Reg 2.5 and DJDC 5.0 (bold print).

**Table 3:** Total marrow irradiation: plan characteristics for Reg 2.5, Reg 5.0 (“regular” delivery with a 2.5 cm and 5.0 cm field width) and DJDC 5.0 (dynamic jaw/dynamic couch delivery with a 5.0 cm maximum field width)

		**Reg 2.5**	**Reg 5.0**	**DJDC 5.0**
PTV	D99 (Gy)	12.2 ± 0.6	11.9 ± 0.6	11.9 ± 0.3
	D1 (Gy)	19.6 ± 0.3	20.1 ± 0.4	19.6 ± 0.3
	**CI**_**95**_	**1.30 ± 0.15**	**1.7 ± 0.23**	**1.47 ± 0.05**
	UI	1.27 ± 0.05	1.34 ± 0.08	1.31 ± 0.07
	TV_95%_	0.781 ± 0.074	0.768 ± 0.037	0.768 ± 0.057
bladder	mean (Gy)	6.8 ± 1.3	8.2 ± 1.1	8.0 ± 1.8
small bowel	mean (Gy)	6.8 ± 1.2	8.2 ± 1.1	7.2 ± 1.0
	D50 (Gy)	6.0 ± 0.9	8.1 ± 3.7	6.6 ± 1.0
esophagus	mean (Gy)	6.7 ± 1.2	9.0 ± 0.5	7.2 ± 1.0
brain	mean (Gy)	8.3 ± 0.6	8.1 ± 1.3	8.7 ± 1.6
	D50 (Gy)	6.9 ± 0.8	7.2 ± 1.7	8.1 ± 0.3
liver	mean (Gy)	**7.6 ± 0.4**	8.3 ± 0.5	**8.5 ± 0.6**
lenses	mean (Gy)	2.4 ± 0.2	2.5 ± 0.3	2.7 ± 0.3
right lung	mean (Gy)	8.0 ± 0.2	8.2 ± 0.3	8.1 ± 0.1
	D50 (Gy)	**6.2 ± 0.3**	6.7 ± 0.3	**6.6 ± 0.1**
left lung	mean (Gy)	8.0 ± 0.1	8.1 ± 0.3	8.1 ± 0.3
	D50 (Gy)	**6.3 ± 0.1**	6.7 ± 0.3	**6.6 ± 0.1**
stomach	mean (Gy)	5.2 ± 0.8	6.6 ± 1.1	5.9 ± 0.7
kidneys	mean (Gy)	**6.1 ± 0.6**	6.0 ± 0.8	**7.1 ± 0.8**
parotid glands	mean (Gy)	**4.2 ± 0.8**	4.2 ± 0.9	**5.8 ± 1.2**
thyroid gland	mean (Gy)	**4.2 ± 0.5**	4.9 ± 0.5	**5.1 ± 0.5**
integral dose	(Gy x l)	631.9 ± 143.4	724.5 ± 140.6	690.0 ± 122.3

Conformity was superior in Reg 2.5 compared to Reg 2.5 and DJDC 5.0 (bold print). Reg 2.5 slightly outperformed DJDC 5.0 in terms of liver, kidney, parotid gland and thyroid sparing and D50 to both lungs (bold print).

Target coverage in the chest wall dropped in all modalities in proximity to the lungs to a level of about 12 Gy. This effect was more pronounced when a 5 cm field width was used. In Figure 4, the equivalent lung sparing and dose fall-off in the target is indicated by light arrowheads.

Conformity was on the same level in all delivery modes for Hemithoracic Irradiation and WAI. In the TMI plans, having the largest and most complex target volumes, conformity was slightly, yet significantly, worse for Reg 5.0 and DJDC 5.0 as compared to Reg 2.5 (see Table 3).

### OAR sparing and integral dose

**Figure 5:**
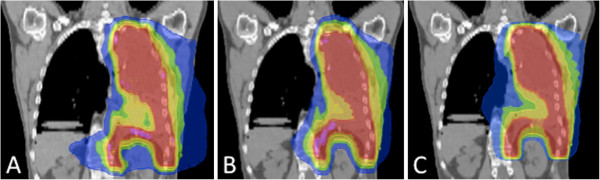
Hemithoracic Irradiation: DJDC 5.0 (C; Dynamic Jaw/Dynamic Couch delivery with a 5.0 cm maximum field width) plans resulted in a reduced dose penumbra compared to Reg 2.5 (A; (“regular” delivery with a 2.5 cm field width) and Reg 5.0 (“regular” delivery with a 5.0 cm field width).

**Figure 6:**
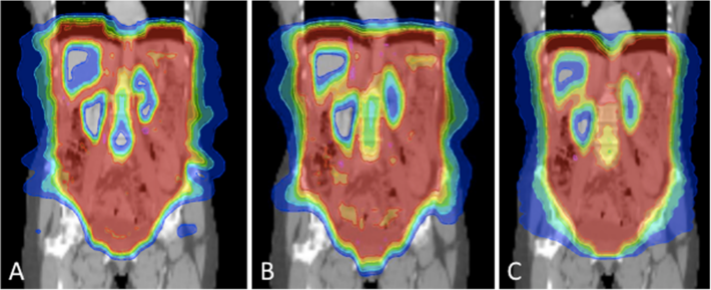
Whole Abdominal Irradiation: Vaginal sparing was better with DJDC 5.0 (C; Dynamic Jaw/Dynamic Couch delivery with a 5.0 cm maximum field width) compared to Reg 2.5 (A; (“regular” delivery with a 2.5 cm field width) and Reg 5.0 (“regular” delivery with a 5.0 cm field width) due to a smaller dose penumbra.

With the dynamic opening and closing of the secondary collimator at the start and end of the target in DJDC 5.0 plans, a smaller craniocaudal dose penumbra compared to Reg 2.5 and Reg 5.0 plans could be realised (see Figures 5 and 6), allowing a better genital sparing in WAI and a sparing of the neck and supraclavicular area in Hemithoracic Irradiation. Consecutively, integral dose was significantly lower in WAI plans when using DJDC 5.0 instead of Reg 2.5 or Reg 5.0. In Hemithoracic Irradiation and TMI, the reduction of integral dose by DJDC compared to Reg 5.0 did not reach statistical significance. In all settings, DJDC 5.0 resulted in a slightly higher integral dose than Reg 2.5 (not significant).

Consequentially, DJDC plans showed a tendency to a higher exposure of organs at risk. In Hemithoracic Irradiation, no significant increase could be detected, with the cardinal organ at risk, the contralateral lung, receiving 6.1 Gy (Reg 2.5) , 6.2 Gy (Reg 5.0) and 6.0 Gy (DJDC 5.0), respectively. Likewise, OAR sparing did not differ significantly in the WAI plans except for a higher bone exposure in Reg 5.0. Most notably, exposure of the liver and both kidneys remained on the same level.

For TMI, mean dose exposure of brain, lenses and lungs remained on a similar level in all delivery modes, while bladder, small bowel and stomach experienced a non-significant dose increase in Reg 5.0 and DJDC 5.0. Kidneys, parotid glands and thyroid gland received a significantly lower dose (∼ 1 Gy) with Reg 2.5 compared to DJDC 5.0, but not to Reg 5.0.

The lungs, however, being the dose-limiting organs, received very similar mean doses of about 8 Gy in all modalities.

## Discussion

This study is the first to show the characteristics of dynamic HT delivery for very large treatment volumes. In all three settings investigated we saw, as expected, a substantial reduction of up to 55% in treatment time when using 5 cm field width instead of 2.5 cm. As a result, plans with Reg 5.0 showed an increased integral dose and larger craniocaudal dose penumbra caused by a larger field width. This effect could be abolished with the use of DJDC delivery which produced fast treatment plans with a sharp craniocaudal dose-falloff. Aside from economic advantages based on a higher patient throughput, shorter treatments primarily increase patient comfort and treatment safety by lowering the risk of intrafractional movement. While treatment time reduction is attractive in every clinical setting, this feature is particularly important for the treatment for very large volumes: For a small target with 1.5 min treatment time, a 50% increase in speed is a small gain compared to the time needed for setup and image guidance. However, speeding up beam-on-time from 30 min to 15 min for large targets constitutes a substantial benefit.

### Hemithoracic irradiation

For patients with Malignant Pleural Mesothelioma, trimodal treatment with extensive surgery including resection of the lung, the visceral and parietal pleura, the pericardium and the diaphragm (extrapleural pneumonectomy, EPP) followed by adjuvant radiotherapy and chemotherapy is a radical treatment approach [11]. The radiooncological target volume consists of the whole former pleural cavity and is in close contact to critical structures such as the contralateral lung, the heart, the liver, the small bowel and the kidney. As pointed out by Allen et al. [12], patients after EPP are more susceptible to lung toxicity than patients without surgery. In a series of 13 patients, 6 developed fatal pneumonitis after fixed-beam IMRT treatment with a mean lung exposure of 15 Gy. HT shows dosimetric advantages over fixed-beam IMRT in terms of dose homogeneity and target coverage as well as OAR sparing. Mean dose to the contralateral lung can be lowered beyond 5 Gy with current HT with 2.5 cm field width [1]. The use of a bigger field width to spare treatment time has not proven clinically feasible because of a large dose penumbra to the neck and the upper abdomen, most notably the liver for right-sided mesothelioma (see Figure 5).

Yet, with DJDC technique, the present study could demonstrate similar dose distributions to “regular” HT. With DJDC 5.0, a drastic reduction of 55% in treatment time could be realised. Considering a treatment schedule of 27 fractions, this would translate into a total reduction of beam-on-time of 2 h 36 min.

Volumetric modulated arc therapy promises very fast IMRT treatments. In the case of 54 Gy hemithoracic irradiation, when comparing fixed-beam IMRT plans to RapidArc® (RA) plans, Scorsetti et al. [13] found equivalent coverage and comparable OAR sparing, while treatment time (from loading of the data to the end of delivery, but excluding time for imaging and patient positioning) was significantly lower for RA (3.7 min vs. 13.4 min). Direct comparison of different planning studies is prone to misinterpretations due to different CTV definitions and OAR contouring; however, compared to our DJDC data, Scorsetti et al. achieved a faster treatment with RA (average 5:10 min vs. 3:42 min, DJDC vs. RA) with comparable dose conformity and sparing of the contralateral lung and kidney. A comparison of liver exposure is impossible as our planning study included more patients with right-sided disease. Target coverage (TV_95%_ 96,7% vs. 93.5, DJDC vs. RA) and sparing of the ipsilateral kidney (mean dose 8.4 Gy vs. 12.9 Gy, DJDC vs. RA) were better in the DJDC plans.

When discussing the technical advances in hemithoracic irradiation after EPP, however, one has to take into account recent data of a randomised UK trial [14] comparing chemotherapy alone to trimodal treatment including EPP in 50 patients. While acute and late radiotherapy side effects in the EPP group were low with only two cases of grade 3 pneumonitis, 1-year overall survival was 52.2% in the EPP group compared to 73.1% in the no EPP group. Among the 16 patients that completed surgery, 4 perioperative deaths occurred. After adjustment for prognostic factors, the hazard ratio for overall survival between the EPP and no EPP group was 2.75. The authors conclude that EPP in a trimodal setting is associated with high morbidity and offers no benefit. Consequentially, EPP has been abandoned in many centers. Even so, the data presented in this study can be applied to other clinical situations such as lung cancer recurrence in the pleural cavity after pneumonectomy or soft tissue sarcoma of the pleural cavity as well.

### Whole abdominal irradiation

Patients with advanced-stage ovarian cancer face a high risk of recurrence in the peritoneal cavity even after maximum cytoreductive surgery and adjuvant chemotherapy, while the risk of metastatic disease outside the abdomen is relatively low [15]. With a 5-year survival rate for patients with FIGO stage III ovarian cancer of 20-25%, long-term prognosis is poor. The integration of Whole Abdominal Irradiation (WAI) into a multimodal consolidation concept has been evaluated with contradictory results, mainly because of high toxicity rates leading to delayed or incomplete radiotherapy [16]. With conventional radiotherapy techniques, delivery of adequate doses to the upper abdomen is impossible due to hepatic and renal toxicity [17]. The introduction of IMRT into clinical routine has opened new possibilities for a less toxic WAI as shown in a phase I trial [2] which might help to increase the locoregional control of locally advanced ovarian cancer. A phase I/II trial using HT is currently recruiting in our department [18].

With Reg 2.5, one WAI treatment fraction of 1.5 Gy takes about 18 minutes. Should WAI be integrated in routine treatment regimes for ovarian cancer, treatment acceleration will become an important issue. Using Reg 5.0 caused a significant drop in target coverage due to the larger field width. DJDC 5.0 plans also showed a significantly, but not as pronounced, loss in coverage. The dynamic jaw component could partly compensate the negative effect of the larger field width. In contrast, DJDC could not counterbalance the higher liver exposure caused by 5.0 cm jaw opening, probably because the gradient to the liver lies more in diagonal direction than in craniocaudal direction. Yet, liver exposure was only increased by about 6% which we believe is not clinically relevant.

Mahantshetty et al. [19] compared WAI with fixed-beam IMRT to RA with three arcs and two isocenters, using 6 MV and 15 MV for both techniques and prescribing 25 Gy to the whole abdomen and a simultaneous integrated boost of 45 Gy to the pelvis. They found comparable target coverage and slightly improved homogeneity for RA. In terms of OAR sparing, 15 MV plans were superior to lower energy plans; 15 MV IMRT and 15 MV RA showed a basic equivalence. Average treatment time was significantly lower for RA (4:8 min for 6 MV and 15 MV RA vs. 18:0 min for 6 MV IMRT and 17:4 min for 15 MV IMRT). As dose prescription differs strongly from our plans, a reliable comparison to our data is difficult. When scaling down our PTV dose to 25 Gy, DJDC would yield a superior liver sparing (mean dose 19.8 Gy vs. 22.4 Gy, DJDC vs. RA).

### Total marrow irradiation

As part of the conditioning regimen before autologuous stem cell transplantation, Total Body Irradiation (TBI) plays a role in the treatment of a broad range of hematological malignancies such as acute lymphoblastic leukemia [20]. However, in the conditioning regimens of other diseases such as Multiple Myeloma, TBI has been abandoned due to extensive toxicity, particularly mucositis, dependence on i.v. nutrition and hospitalisation time [21,22]. The standard TBI technique uses large ap/pa and lateral fields while blocking the lungs and an electron boost to the thoracic wall and is associated with substantial toxicity. While restriction of the target volume to the bone marrow with conventional techniques is still associated with substantial toxicity [23], modern IMRT techniques promise a further reduction of OAR exposure [24]. Technical and clinical feasibility of Total Marrow Irradiation (TMI) using HT have been demonstrated by Wong et al. in 2006 [25]. In a phase I dose escalation trial, TMI was investigated as part of a tandem autologuous stem cell transplantation regimen for patients with Multiple Myeloma. Dose-limiting toxicity was determined at 18 Gy. Up to a total dose of 16 Gy, TMI was tolerated well with a low incidence of grade 3 nonhematological toxicities [26]. One major drawback of advanced TMI techniques are, however, the long treatment times required for highly conformal dose delivery. For state-of-the art HT, treatment times of 20–40 min are reported, depending on the chosen field width [27].

For technical reasons, the maximum target length for HT is limited to 160 cm. Like most other groups using HT for TMI, we would address this problem by treating the lower extremities with ap/pa fields on a linear accelerator.

In the present study, Reg 2.5, Reg 5.0 and DJDC 5.0 could deliver the same dose to the target. The lungs as the dose-limiting organs caused a dose fall-off to about 12 Gy particularly in Reg 5.0 and DJDC 5.0 delivery, which is probably due to the larger maximum field width. This phenomenon can also be seen in the DVHs published by Han et al. [27]. Regarding a prescribed dose of 18 Gy, this appears to be an unacceptable underdosage at first glance. However, this is the dose level the patient would have received with conventional TBI to the whole target. Thus, with both modalities, 99% of the PTV received the conventional TBI dose or more, and 76% received 17.1 Gy or more.

Some OAR were exposed to a significantly higher mean dose with DJDC 5.0 compared to Reg 2.5, in particular the liver, the kidneys, the parotid glans and the thyroid gland. As the Reg 5.0 plans showed no significant dose increase in those organs, this might be an effect of the dynamic component. Yet, OAR exposure was raised by approximately 1 Gy which is probably not clinically relevant.

Current HT is often criticised because of long treatment times and challenged by volumetric arc modulated techniques that promise faster treatments. Very recently, Han et al. [27] showed in a plan comparison study for TMI a reduction of average beam-on time from 1122 sec to 628 sec when using RA instead of HT with 5 cm field width. In addition, mean OAR dose could be lowered significantly in some organs such as the brain, the right kidney, the optic nerves and the thyroid gland. However, mean dose to the intestines as well as D10 (dose covering 10% of the volume) for the intestines, both lungs and the stomach were significantly higher in RA plans. On high dose levels (D80), no difference in lung exposure could be detected. In addition to beam-on-time, time for isocenter setup is needed for each arc field in RA technique. Thus, the authors estimate that total treatment time for RA and HT would be comparable. Our results with an average treatment time of 1105 sec for TMI are in line with their data.

One feature of DJDC technique is its ability to diminish the craniocaudal dose penumbra and consecutively the integral dose as demonstrated for nasopharyngeal cancer [28]. Contrary to expectations, in the present study, a significantly lower integral dose with DJDC as compared to Reg 5.0 could only be demonstrated for WAI. In all other settings, integral dose did not differ significantly between Reg 2.5, Reg 5.0 and DJDC 5.0, which is probably due to crucial differences in PTV shape: For nasopharyngeal cancer, the relation of longitudinal to lateral extension is much smaller than for the large volumens investigated in this study, giving the size of the longitudinal penumbra a bigger influence. In addition, the main dose gradient in nasopharyngeal cancer treatment (i.e. towards the myelon) is situated more or less on the same coronal plane. In contrast, the gradients in Hemithoracic Irradiation, WAI in TMI vary in location and cross the coronal planes, e.g. along the lower rim of the liver. In such a setting, the bigger maximum field width in DJDC causes a bigger lateral dose penumbra and outweighs the gain achieved by the reduced craniocaudal penumbra.

## Conclusions

The HT treatment of large volumes such as the hemithorax, the abdominal cavity or the complete bone marrow can be sped up substantially by DJDC, while maintaining excellent target coverage, OAR sparing and sharp dose gradients above and below the target. Especially in the case of targets with very large craniocaudal extensions such as Total Marrow Irradiation, it constitutes a convenient and safe treatment modality. However, for the time being, dynamic HT is still under development and not available for clinical routine yet.

## Abbreviations

DJDC: Dynamic jaw/dynamic couch; HT: Helical tomotherapy; IMF: Intensity modulation factor; IMRT: Intensity-modulated radiotherapy; OAR: Organ at risk; MLC: Multi-leaf-collimator; TMI: Total marrow irradiation; WAI: Whole abdominal irradiation; D_1/99_: Dose to 1%/99% of the target volume; CI_95_: Conformity index total volume covered by the 95% isodose divided by the volume of the PTV covered by the 95% isodose; UI: Uniformity index dose covering 5% of the PTV divided by the dose covering 95% of the PTV; TV_95%_: Volume covered by 95% of the prescribed dose divided by the PTV volume; EPP: Extrapleural pneumonectomy; RA: RapidArc®.

## Competing interests

The authors have no competing interests to declare.

## Authors’ contributions

SK was responsible for drafting the manuscript. SB did the treatment planning and plan evaluation. KS and SL were responsible for data and software management. KH contributed to the revision of the manuscript. JD as head of the Department of Radiation Oncology has given final approval to the article. FS as head of the Tomotherapy research group at the Department of Radiation Oncology was responsible for conception and design of the study, treatment planning and revision of the manuscript. All authors read and approved the final manuscript.
